# Computed Tomography-Image-Based Glioma Grading Using Radiomics and Machine Learning: A Proof-of-Principle Study

**DOI:** 10.3390/cancers17020322

**Published:** 2025-01-20

**Authors:** Melike Bilgin, Sabriye Sennur Bilgin, Burak Han Akkurt, Walter Heindel, Manoj Mannil, Manfred Musigmann

**Affiliations:** Clinic for Radiology, University of Münster and University Hospital Münster, Albert-Schweitzer-Campus 1, DE-48149 Muenster, Germanymanfred.musigmann@gmx.de (M.M.)

**Keywords:** glioma grading, neuroimaging, machine learning, radiomics, CT, artificial intelligence

## Abstract

Background/Objectives: In recent years, numerous studies have been published on determining the WHO grade of central nervous system (CNS) tumors using machine learning algorithms. These studies are usually based on magnetic resonance imaging (MRI) and sometimes also on positron emission tomography (PET) images. To date, however, there are virtually no corresponding studies based on routinely generated computed tomography (CT) images. The aim of our proof-of-concept study is to investigate whether machine learning-based tumor diagnosis is also possible using CT images. Methods: We investigate the differentiability of histologically confirmed low-grade and high-grade gliomas. Three conventional machine learning algorithms and a neural net are tested. In addition, we analyze which of the common imaging methods (MRI or CT) appears to be best suited for the diagnostic question under investigation when machine learning algorithms are used. For this purpose, we compare our results based on CT images with numerous studies based on MRI scans. Results: Our best-performing model includes six features and is obtained using univariate analysis for feature preselection and a Naive Bayes approach for model construction. Using independent test data, this model yields a mean AUC of 0.903, a mean accuracy of 0.839, a mean sensitivity of 0.807 and a mean specificity of 0.864. Conclusions: Our results demonstrate that low-grade and high-grade gliomas can be differentiated with high accuracy using machine learning algorithms, not only based on the usual MRI scans, but also based on CT images. In the future, such CT-image-based models can help to further accelerate brain tumor diagnostics and to reduce the number of necessary biopsies.

## Simple Summary

In recent years, numerous studies have been conducted to determine the WHO grade of central nervous system tumors using machine learning algorithms. These studies are typically based on magnetic resonance imaging (MRI) and sometimes positron emission tomography images. However, to date, there are almost no such studies based on routinely acquired computed tomography (CT) images. In our proof-of-concept study, we show that machine learning-based tumor diagnostics is also feasible based on routinely acquired CT images. The models developed in our study show comparably high accuracies to those based on MRI images. In the future, such CT-image-based models can help to further accelerate brain tumor diagnostics and to reduce the number of necessary biopsies.

## 1. Introduction

Gliomas represent 25.5% of all primary central nervous system (CNS) tumors, and are the second most common brain tumors in adults [1,2]. Annually, approximately six cases of CNS gliomas are diagnosed per 100,000 people worldwide [3]. Gliomas, which are known to originate from glial cells, can be divided into non-diffuse and diffuse gliomas, with non-diffuse gliomas exhibiting a more circumscribed growth pattern, while diffuse gliomas show infiltrative growth [4]. The criteria for the classification of CNS tumors are regularly revised by the World Health Organization (WHO). Before the release of the 2007 version of the WHO classification of CNS tumors, gliomas were characterized by their histology [5]. According to the WHO classification of 2016, diffuse gliomas were histopathologically divided into astrocytic, oligodendroglial or oligoastrocytic tumors. Astrocytomas are of astrocytic origin, and oligodendrogliomas are known to originate from oligodendrocytes [6]. Oligoastrocytomas, on the other hand, were described as showing both astrocytic and oligodendroglial components [7]. However, this subtype no longer exists according to the current WHO classification. According to the WHO classification of 2016, the tumors were described as exhibiting different malignancy grades: II (low-grade), III (anaplastic) and IV (glioblastoma) [4,8]. Tumor grades III and IV are often grouped together as high-grade gliomas, resulting in the important distinction between low-grade gliomas (LGGs) and high-grade gliomas (HGGs). However, LGGs generally progress over time and transform into HGGs [9,10]. In addition to the histological criteria already mentioned, molecular criteria were introduced in the 2016 version of the WHO classification of CNS tumors to improve diagnosis and treatment plans [11,12]. In the latest, fifth version, published in 2021, the use of molecular diagnostics has been further expanded. New tumor entities have been introduced, some of which are based on new diagnostic methods, such as DNA methylome profiling to differentiate tumors with overlapping histological features; in addition, there have also been some changes in nomenclature. Some tumors have also been recategorized; for instance, adult-type diffuse gliomas are now categorized as diffuse astrocytoma, IDH-mutant; oligodendroglioma, IDH-mutant; and 1p/19q-codeleted and glioblastoma, IDH-wildtype [13,14,15].

The exact determination of the tumor’s subtype and WHO grade is very important for the further treatment of patients and their prognosis. The treatment plan differs between LGGs and HGGs. Treatment options for LGGs include surgery and close clinical and imaging follow-up [16,17]. Patients with a high risk of early malignant transformation undergo radiation therapy after surgical intervention [18]. The standard treatment for HGGs consists of maximal surgical resection followed by radiation and adjuvant chemotherapy, such as temozolomide [19,20].

Prognosis and survival also depend on WHO grade/tumor subtype, with an increased survival probability with a lower tumor grade, younger age at diagnosis and IDH-mutated, 1p/19q-codeleted molecular subtype [21]. For example, the median survival time of patients with IDH-mutated WHO grade II gliomas is more than 10 years [22]. Furthermore, the prognosis of IDH-wildtype anaplastic astrocytomas is worse (approx. 20 months) in comparison with IDH-mutated anaplastic WHO grade III astrocytomas (approx. 65–81 months) [23,24]. To be able to plan the best possible treatment for the patient, it is therefore very important to determine the WHO grade/tumor subtype and to reliably distinguish between low- and high-grade gliomas. Imaging techniques such as computed tomography (CT) are crucial for the diagnosis of brain tumors and for assessing treatment and prognosis [25]. Magnetic resonance imaging (MRI) is the most sensitive imaging modality for diagnosing tumors of the CNS; however, it is difficult to differentiate tumor entities based on imaging methods alone [26].

In recent years, there have been many studies analyzing the value of radiomics-based machine learning algorithms to support such diagnostic challenges. Radiomics seeks to extract quantitative and reproducible information from medical diagnostic images (MRI, CT or PET), including complex patterns that are difficult for the human eye to recognize or measure [27,28]. In combination with bioinformatics tools, models can be created that could increase the accuracy of diagnostic, prognostic and predictive methods [27]. As an example, a recent study demonstrated that radiomics-based machine learning is able to differentiate between pseudoprogression and true progression in HGGs, potentially reducing the use of invasive histopathology in the future [29]. Biopsy remains the gold standard for determining tumor grade/subtype. However, invasive biopsies are associated with risks such as impaired consciousness, seizures, brain swelling and hemorrhaging, as well as mortality [30], and may also be impractical in certain clinical scenarios. If, in the future, radiomics-based machine learning can replace time-consuming laboratory tests, such new methods will also help to further accelerate the diagnostic process. Li et al. used MRI scans and a radiomics-based method to predict glioma subtypes as defined by tumor grade, among other characteristics [31]. MRI-based machine learning also helps to non-invasively differentiate between molecular alterations in gliomas, such as 1p/19q codeletion [32]; to distinguish between HGGs and brain metastases [33]; or to predict the possibility of complete resection of meningiomas [34].

The important diagnostic questions both of the exact determination of the WHO grade/tumor subtype of CNS tumors using radiomics-based machine learning algorithms [35,36,37,38,39,40] and the binary differentiation between LGGs and HGGs, have already been investigated in various studies [35,41,42], including four meta-studies [43,44,45,46]. For example, Chen et al. focused on differentiating low-grade astrocytomas and anaplastic astrocytomas using contrast-enhanced T1 MRI with an AUC of 0.825 [41]. Sengupta et al. achieved glioma grading with low classification errors on T1 perfusion MRI using a machine learning algorithm [47]. Liu et al. investigated the preoperative differentiation of supratentorial non-enhancing LGGs from HGGs using diffusion tensor MRI and perfusion-weighted imaging [48].

Almost without exception, these studies are based on MRI scans. In only a few studies, PET images [46] or other techniques, such as fluorescence imaging or stimulated Raman histology imaging [49], are used. In contrast, we could find virtually no studies in which the differentiation of LGGs and HGGs, or the determination of the tumor grade of CNS tumors, per se, was analyzed with machine learning algorithms based on CT images, although CT images are almost always available for affected patients. The question arises as to whether CT images are not just as suitable, or even better, for this important diagnostic issue.

The aim of our study is therefore to analyze whether it is also possible to differentiate between LGGs and HGGs using machine learning algorithms based on CT images. We analyze how accurately the two entities mentioned can be distinguished using machine learning algorithms based on CT images. In the Section 4, we compare our results with corresponding studies based on MRI scans to gain an impression of which of the imaging techniques (MRI or CT) appears to be more suitable for the diagnostic question investigated in this study.

## 2. Materials and Methods

Our study was performed in compliance with the Declaration of Helsinki and approved by the local ethics committee (Ärztekammer Westfalen Lippe and University of Münster, 2021-596-f-S). Due to its retrospective nature, written informed consent was waived (Ärztekammer Westfalen Lippe and University of Münster). We reviewed the Picture Archiving and Communication System (PACS) of our hospital to identify cases of LGGs and HGGs recorded between 2008 and 2020. The imaging data were acquired using dual-energy CT scanners, specifically the Somatom Force and Somatom Definition AS models from Siemens Healthineers, Germany. CT images were downloaded in DICOM format with a pseudonymized DICOM header. We used contrast-enhanced CT imaging for navigation, with a typical voxel size of 512 × 512 and a slice thickness of 1 mm. All CT images were acquired using the same CT protocol. Contrast-enhanced CT images were available for 141 patients with a histopathological confirmed glioma. According to their WHO grade, we differentiated the gliomas as LGGs and HGGs. We excluded 15 patients due to very small tumor size with little contrast enhancement and without precise detectability on CT-imaging, or due to extensive edema. We also excluded patients with a history of glioma, who had undergone surgical tumor resection, with a present tumor recurrence and a difficulty to segment the recurrent tumor part due to a large parenchymal defect. Finally, 126 patients were included. Of these, 57 had an LGG, and the remaining 69 patients had an HGG.

### 2.1. Radiomics

**Figure 1:**
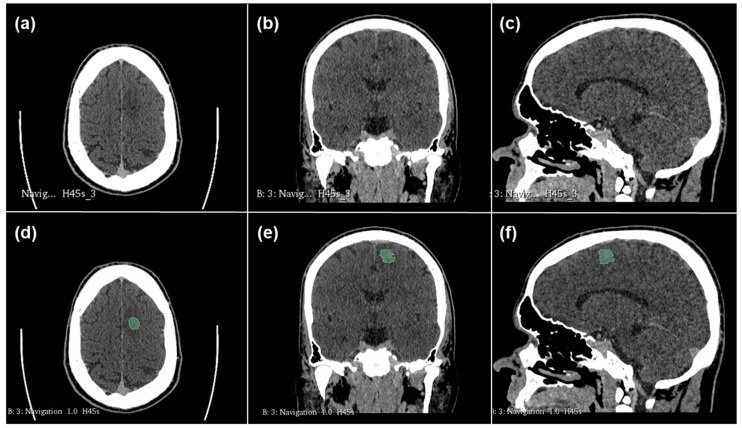
Case of low-grade glioma (LGG). Patient aged 45 years old with oligodendroglioma localized in left frontal lobe. Images (**a**–**c**): original CT images before segmentation. Images (**d**–**f**): semi-automatic segmentation with 3D Slicer (areas marked in green).

**Figure 2:**
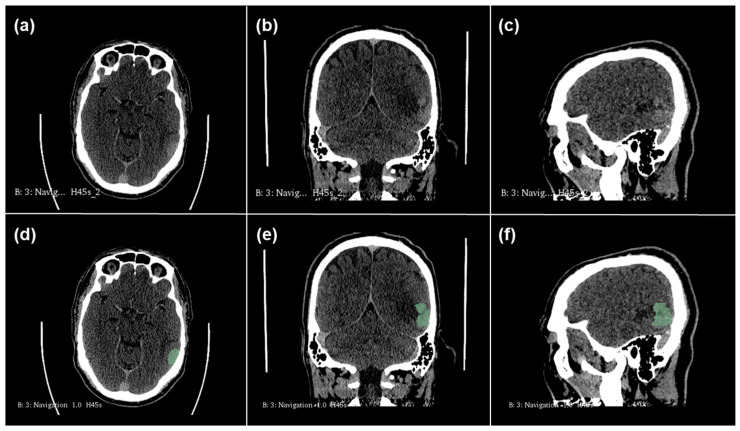
Case of high-grade glioma (HGG). Patient aged 49 years old with astrocytoma localized in left parietal lobe. Images (**a**–**c**): original CT images before segmentation. Images (**d**–**f**): semi-automatic segmentation with 3D Slicer (areas marked in green).

All segmentations were performed with the semi-automatic segmentation tool of the 3D Slicer open-source software platform (version 4.10, www.slicer.org, accessed on 16 December 2021), using the Segmentation Wizard plugin. The contrast-enhancing parts of the tumor on the contrast-enhanced CT images were segmented by an experienced radiologist with 2 years of neuroradiology training, and all segmentations were reviewed by a board-certified neuroradiologist, with professional consensus employed for challenging cases of segmentation. As an example, Figure 1 shows the CT images of a patient with an LGG, and Figure 2 shows the corresponding images of a patient with an HGG. The partial images a to c show the CT images before segmentation. The semi-automatic segmentation performed with the 3D Slicer is shown in green in the partial images d to f. Using the open-source PyRadiomic software (https://pyradiomics.readthedocs.io/en/latest/, accessed on 15 January 2025), we calculated a total of 107 different radiomics features. The PyRadiomic software is available as an implementable plugin into the 3D Slicer platform. The radiomics features can be divided into 7 different feature classes: 18 first-order statistics, 14 shape-based features, 24 gray-level co-occurrence matrices, 16 gray-level run-length matrices, 16 gray-level size-zone matrices, 5 neighboring gray-tone difference matrices and 14 gray-level dependence matrices. In addition, our database included patient age at diagnosis, gender and tumor subtype as further features. All features were z-score transformed and subjected to a 95% correlation filter to account for possible redundancy among the features.

### 2.2. Statistical Analysis

Our study cohort consisted of 126 patients. The patients were allocated to training data and independent test data at random. Specifically, a stratified 4:1 ratio (training data: 101 patients, test data: 25 patients) was used, with a balanced distribution of LGGs and HGGs between the two samples. The demographic and histopathological distributions of the training data, the independent test data and the total study cohort are summarized in Table 1.

Feature preselection and subsequent model construction were performed using the training data. Model performance was calculated based on the independent test data. A challenge in many studies on brain tumors is the often quite small number of cases in the available study cohorts. Our study cohort, consisting of 126 patients, is also comparatively small. To solve this challenge, we developed a technique that can be used to check model stability and at the same time reduce the probability of overfitting. For this purpose, we performed the division of the data into training and independent test data, the preselection of features, the model development, and the testing of the final models 100 times each. Subsequently, we checked the model’s stability and its performance over these 100 cycles. This also allowed us to determine reliable confidence intervals for all performance metrics. Furthermore, all models were developed with an increasing number of features. Based on the results obtained with the independent test data, we could thus determine the model complexity at which overfitting begins. Overall, the technique described allows the minimization of random effects associated with data partitioning and the likelihood of overfitting, while also verifying model stability.

**Figure 3:**
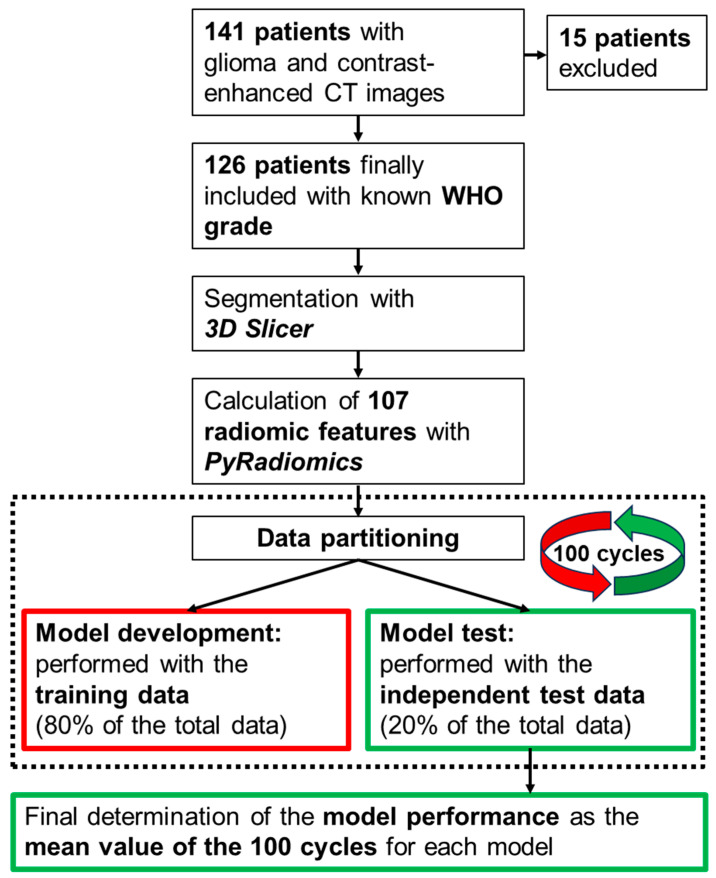
Flowchart describing the methodological approach to distinguish between LGGs and HGGs based on contrast-enhanced CT images. Three different feature preselection algorithms are tested, in combination with four different machine learning algorithms for subsequent model development (see text for a detailed description).

The exact algorithms used for the feature preselection and model training to discriminate between LGGs and HGGs, the hyperparameters included in the models and their optimization, and other important points for model development are discussed in detail below. Before this, Figure 3 shows a flowchart of the methodological approach used to develop each individual model. First, the study cohort was collected and the radiomic features were calculated. The steps shown in the dashed box were subsequently repeated 100 times, using new data split into training and independent test data in each cycle. Based on the training data sets, the feature preselection and the complete model optimization were repeated in each cycle. The hyperparameters/coefficients included in the models were optimized using grid search and repeated 10-fold cross-validation. The models were optimized by maximizing the area under the curve (AUC) of the receiver-operator characteristic (ROC). In each of the 100 runs, the individual model performance was determined based on the associated independent test data. At the very end (last field in Figure 3), the model performance was determined as the mean value based on the 100 cycles.

We performed our statistical analysis using R software (version 4.1.2). To distinguish LGGs from HGGs, we tested three different feature preselection methods, in combination with four different machine learning algorithms, to differentiate the two entities. This resulted in a total of 12 individual models. We used the “caret” package in R to develop each of these models. The development of each of our individual models thus consisted of two main steps: Step 1: the preselection of features suitable for distinguishing between HGGs and LGGs; and Step 2: the subsequent training and testing of a model to distinguish between LGGs and HGGs based on the features identified in the previous step.

Step 1: As already explained, we used three different approaches to preselect the features. In detail, we determined the features based on univariate analyses, the Naïve Bayes algorithm and Ridge regression. We used the “nb” method from the caret package for the Naive Bayes algorithm, and the “glmnet” method for Ridge Regression. In the case of univariate analyses, the most discriminatory features were determined based on the Akaike information criterion (AIC). In the case of the two multivariate methods (Naive Bayes algorithm and Ridge regression), the “VarImp” function in R was used for feature preselection. Depending on the machine learning algorithm used, the “VarImp” function uses different methods to estimate the contribution of each variable to the model. In some machine learning algorithms, the importance of individual model features can be determined, for example, by calculating the performance of the model (AUC) with and without the inclusion of the feature. The difference between the two performance values obtained in this way determines the performance gain resulting from this feature. The feature that causes the highest performance loss when removed from the model is the most important, and so on. In a random forest approach, on the other hand, the prediction accuracy for each tree can be determined on the out-of-bag portion of data. The same process is then carried out after permuting each predictor variable. Finally, the difference between the two accuracies is averaged across all trees. Readers interested in a deeper exploration of the methodology of the “VarImp” function in R can find more information in the R documentation regarding this method. We conducted all subsequent model developments (separated according to the three different feature preselection methods) using the most important 1 to 10 features. In each case, a univariate model was developed first, which only contained the most important feature. The second most important feature was subsequently added to this model, followed by the third most important feature, etc. We used an increasing number of features to determine the number of features from which the models began to become overfitted. In this way, the optimal number of features to be used could be determined.

Step 2: To differentiate between LGGs and HGGs, four different machine learning algorithms were subsequently tested. Specifically, we trained a Naive Bayes algorithm, a linear discriminant analysis (LDA), a random forest algorithm, and a neural network. In detail, we used the methods “nb” (Naive Bayes), “lda” (linear discriminant analysis), “rf” (random forest) and “nnet” (neural network) from the caret package for this purpose. The method “nnet“ can be used to train a feed-forward neural network with a single hidden layer. In addition to the coefficients, the individual models also contain hyperparameters. When training the models, it is crucial that these coefficients/hyperparameters are optimized using suitable techniques. We optimized the hyperparameters included in our various models using the grid search technique. Specifically, grid search was used to determine the Ridge regression parameter “lambda” (L2 regularization parameter, which was only used during feature preselection), the two parameters “mtry” and “ntree” (number of randomly selected predictors at each split, number of trees) in the random forest algorithm, and the parameters “size” and “decay” (number of units in the hidden layer, parameter for weight decay) in the neural network. When using the “glmnet” method (used only for feature preselection), the second hyperparameter, alpha, was fixed to achieve a Ridge regression and not an elastic net. In the neural network, the maximum number of iterations was set to 1000 (parameter “maxit”), which was sufficient for the optimization. All other parameters included in the machine learning algorithms mentioned were not optimized by grid search, but were automatically optimized by the caret package. When training the models, a 10-fold cross-validation with 10 repetitions each was used to determine/optimize the model parameters (method = “repeatedcv”, number = 10, repeats = 10). All models were optimized by maximizing the area under the curve (AUC) of the receiver-operator characteristic (ROC). As described above, each individual model was fully developed 100 times and subsequently tested. For each of these runs, the data partitioning was carried out again, resulting in 100 different training samples and 100 samples with independent test data. All performance values were determined, based on the associated independent test data, as the average of 100 runs. At the very end, we once again tested the significance of all the features included in our overall best model. For this purpose, we once again conducted both univariate and feature importance analyses to ensure the univariate and multivariate significance of all the features included in the final model.

In the context of our publication, only a very superficial presentation of the respective methodologies of the machine learning algorithms used can be given. The Ridge regression used in our study is an algorithm that is related to ordinary regression methods such as logistic regression, but additionally includes a penalty/regularization parameter to avoid overfitting. The Naive Bayes algorithm is a classification method based on Bayes’ theorem. In very simplified terms, suitable features are determined to distinguish between the different classes (e.g., LGGs and HGSs). It is important to note that the Naive Bayes algorithm assumes that the occurrence of a feature within a class is completely uncorrelated with the occurrence of the other features within that class. A typical application of the Naive Bayes algorithm is a spam filter for e-mails. In contrast, the LDA tries to distinguish between two or more classes by searching for suitable linear combinations of features. Similarly to regression analyses, LDA also attempts to express a dependent variable (the class to be predicted) as a linear combination of suitable other features. The random forest algorithm uses a method similar to that of a decision tree to distinguish the classes to be separated. In contrast to a single decision tree, however, the random forest algorithm uses an entire “forest” of decision trees for this purpose.

As described above, we trained all the machine learning models in our study using the caret package in R, based on the methods “glmnet”, “nb”, “lda”, “rf” and “nnet”. The exact names of the hyperparameters contained in these methods (which were optimized by grid search, as described) have also already been provided. Readers interested in even more precise information on the specifications of the machine learning algorithms used in our study can find this information in the documentation for the caret package in R. Further information on the caret package has also been published by Kuhn [50]. For more information on machine learning in general, we refer the reader to the relevant literature (e.g., [51,52,53]).

## 3. Results

As described, we tested three different algorithms for feature preselection. Table 2 summarizes the 10 most important features obtained with the three different feature preselection methods. The four features “gldm.DependenceEntropy”, “glcm.Correlation“, “glcm.Idmn“ and “glrlm.GrayLevelNonUniformityNormalized“ were selected by all three algorithms. It should be noted that the 100 cycles per model resulted in different most important features per cycle, due to the different training data sets. The table summarizes the most important features that were determined during the 100 runs in total.

**Figure 4:**
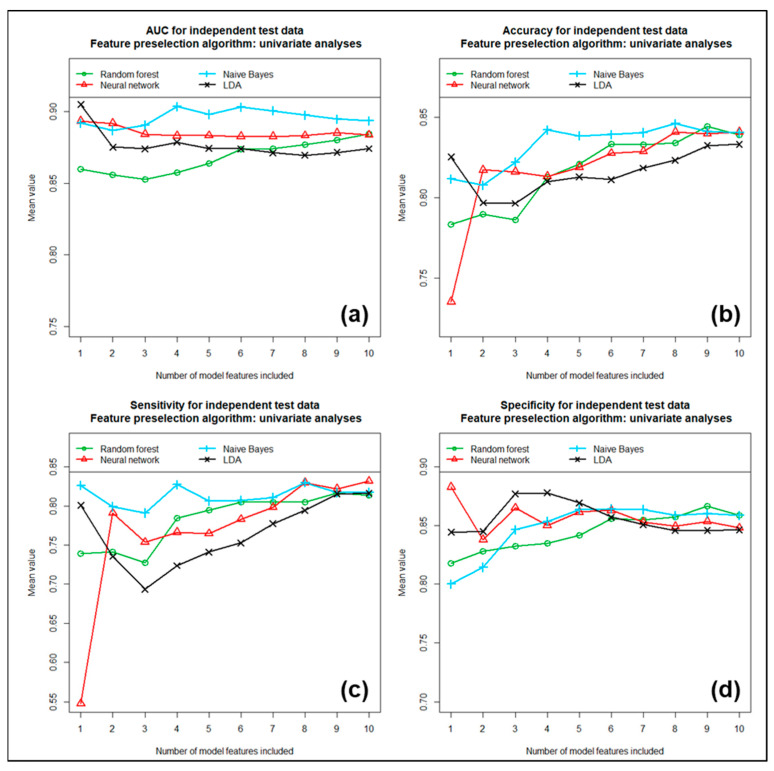
(**a**) AUC, (**b**) accuracy, (**c**) sensitivity and (**d**) specificity for independent test samples using univariate analysis for feature preselection. All values are calculated as means of 100 repetitions.

**Figure 5:**
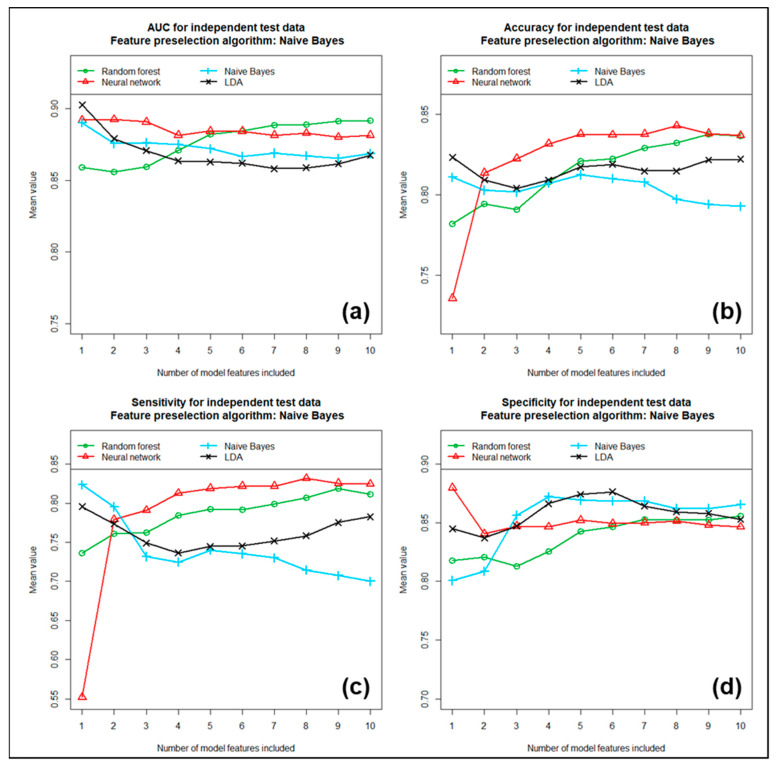
(**a**) AUC, (**b**) accuracy, (**c**) sensitivity and (**d**) specificity for independent test samples, using Naïve Bayes algorithm for feature preselection. All values are calculated as means of 100 repetitions.

**Figure 6:**
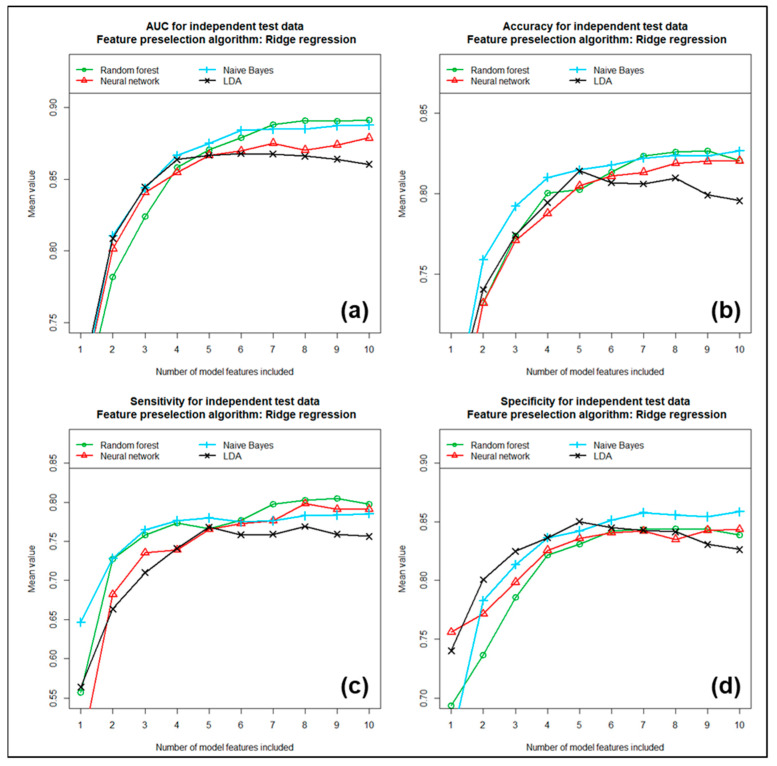
(**a**) AUC, (**b**) accuracy, (**c**) sensitivity and (**d**) specificity for independent test samples, using Ridge regression for feature preselection. All values are calculated as means of 100 repetitions.

Each of these three feature preselection methods was subsequently tested in combination with each of the four previously mentioned machine learning algorithms for its ability to discriminate between LGGs and HGGs. We included the first 1 to 10 most important of the previously determined features in the models. The performance achieved with these individual models is shown in Figure 4, Figure 5 and Figure 6 as a function of the number of features included.

Figure 4 shows the mean AUC (partial image a), mean accuracy (partial image b), mean sensitivity (partial image c) and mean specificity (partial image d) obtained with the four different machine learning algorithms, tested as a function of the number of features included for the case where feature preselection was performed using univariate analyses. In the second case, where feature preselection was performed using the Naïve-Bayes algorithm, the corresponding results are summarized in Figure 5, and finally, in Figure 6, the results are summarized for the third case, in which feature preselection was performed using Ridge regression. All three different methods for feature preselection resulted in high and similar discriminatory power values. For most models, the number of features included had only a limited effect on discriminatory power. Using more than six features did not significantly further increase the discriminatory power in most cases. When comparing the four machine learning algorithms tested for model development, the Naïve-Bayes approach often performed best overall in terms of the tested metrics, while the LDA approach often performed worst. The random forest approach and the neural network achieved mid-range performance results. In terms of AUC and accuracy, the combination of univariate analyses followed by a Naïve Bayesian approach performed best overall. Using the six most important features, this model yielded a mean AUC of 0.932, a mean accuracy of 0.862, a mean sensitivity of 0.849, a mean specificity of 0.874 and a mean Cohen’s kappa of 0.722, based on the training data sets. Here, the sensitivity describes the proportion of correctly predicted high-grade gliomas (HGGs) and, correspondingly, the specific proportion of correctly predicted low-grade gliomas (LGGs). Due to the method used for modeling, which is based on 100 repetitions each, these mean values turned out to be quite independent of the data samples used. Using the independent test data sets resulted in performance values that were only slightly lower than those obtained with the training data. Here, we received a mean AUC of 0.903, a mean accuracy of 0.839, a mean sensitivity of 0.807, a mean specificity of 0.864 and a mean Cohen’s kappa of 0.673. Table 3 summarizes the results obtained with the training data sets compared to the corresponding results obtained with the independent test data sets. The numbers in brackets in the table indicate the 95% confidence intervals. It is obvious that the final model exhibits a high and, at the same time, very stable discriminatory power. We also determined the order of importance of the individual features included in the multivariate models using feature importance analyses. These analyses yielded the following ranking, in descending order of importance (see also Table 2): (1.) “gldm.DependenceEntropy”, (2.) “glrlm.RunEntropy”, (3.) “glcm.Correlation”, (4.) “glszm.ZoneEntropy”, (5.) “glrlm.GrayLevelNonUniformity Normalized” and (6.) “glcm.Idmn”. The “glrlm.GrayLevelNonUniformityNormalized” feature is negatively correlated with WHO grade. In contrast, the other five features show an increasing probability of HGG with increasing feature value. All six features exhibited very high univariate discriminatory power, which, based on the entire study cohort, was between AUC = 0.833 and 0.898. In addition, the multivariate significance of each feature was checked again using feature importance analysis.

## 4. Discussion

Our results show that machine learning models based on CT images are able to distinguish between LGGs and HGGs with high accuracy. Our best-performing model containing the six most important features was developed using a combination of univariate analyses and a naïve Bayesian approach for feature preselection and model construction, respectively. Based on the independent test data, we obtained a mean AUC of 0.903, a mean accuracy of 0.839, a mean sensitivity of 0.807, a mean specificity of 0.864 and a mean Cohen’s kappa of 0.673.

As already mentioned, the distinction between LGGs and HGGs is of great importance for further treatment planning and prognosis. However, the question arises of whether the CT image-based discrimination of LGGs and HGGs performed in our study is more or less accurate compared to similar machine learning approaches that are based on other medical imaging techniques, such as MRI or PET. To gain an impression in this respect, we compared our results with corresponding results from a total of three meta-studies. The first of these meta-studies was performed by Wang et al. [43]. It was based on 15 individual studies in which radiomics based on MRI scans was used to differentiate between LGGs and HGGs. This meta-study resulted in a pooled sensitivity of 0.92 [0.89, 0.95], a pooled specificity of 0.89 [0.85, 0.92] and an AUC of the summary receiver operating characteristic of 0.91 [0.88, 0.93]. The values in parentheses again indicate the 95% confidence intervals. A second meta-study, which also included studies involving MRI-based machine learning algorithms, was conducted by De Maria et al. [44]. Most of the individual studies included used conventional machine learning algorithms, such as logistic regression or support vector machines. A few studies were also based on deep learning. This meta-study yielded a sensitivity of 0.84 [0.78, 0.89] and a specificity of 0.91 [0.86, 0.94] for the differentiation between LGGs and HGGs. In contrast, Sun et al. conducted a meta-study in which they included a total of 33 sub-studies, all of which used deep learning algorithms [45]. The authors determined a pooled sensitivity of 0.93 [0.89, 0.96], a pooled specificity of 0.94 [0.89, 0.96] and an AUC of 0.98 [0.96, 0.99]. The studies included were, again, almost without exception, based on MRI scans. Fluorescent imaging was used in one included study and stimulated Raman histology imaging in another. These two sub-studies resulted both in below-average performance values compared to the other MRI-based studies included in this meta-study.

In summary, the three meta-studies listed [43,44,45], which are mainly based on MRI scans, showed a slightly higher discriminatory power, on average, compared to our results based on CT images (see Table 3). Here, the studies based on deep learning algorithms performed even slightly better than the studies in which conventional machine learning algorithms were used. It should be noted that we also searched for meta-studies based on PET images, but so far, we have not been able to find a radiomics-based PET meta-study involving machine learning algorithms. However, in a PET-based meta-analysis conducted by Delgado et al. on studies not using radiomics-based machine learning [46], the average accuracies in distinguishing between LGGs and HGGs were slightly lower than the accuracies found in our study.

At this point, it must be clearly stated that a valid comparison with our results based on CT images is only possible to a limited extent. The study cohorts used in the individual sub-studies are very heterogeneous in terms of cohort size, the composition of the tumor grades included, the imaging techniques used, different MRI sequences and many other influencing factors. Another important point to note is that the individual sub-studies used different definitions for LGGs and HGGs. In some sub-studies, grade 3 gliomas were classified as HGGs, and in other studies, as LGGs. Finally, it should also be noted that the performance values given in the sub-studies are of varying reliability. For example, in contrast to the approach we used, in some studies, the performance values were not determined based on independent test data.

As described, numerous previous studies analyzing the differentiation of gliomas are based on the use of MRI scans. Contrary to these studies, our study is based on contrast-enhanced CT images that are performed routinely. To the best of our knowledge, there are comparatively few other studies that use CT images to differentiate brain tumors in combination with radiomics-based machine learning. Recently, a single corresponding study was published by Maskani et al. [54]. The discriminatory power values obtained in this study in the discrimination of LGGs and HGGs were even higher than the corresponding values obtained in our study. However, it should be noted that the cohort used in this study comprised 62 patients, and was therefore comparatively small. In addition, the discriminatory power values were determined using cross-validation, and were not based on completely independent test samples. A second study using CT imaging and radiomics-based machine learning was performed by Mărginean et al. [55]. They investigated the differentiability of gliomas from brain metastases in the peritumoral zone. This and other studies demonstrate that, in addition to MRI scans, CT images can also offer great added value in the field of machine learning-based diagnostics.

## 5. Limitations

There are some limitations to our study. As a first limitation, it should be noted that our study was a retrospective study conducted at a single institution. Secondly, our study cohort comprised 126 patients, which is comparatively small. Due to the comparatively small number of cases in our study cohort, we decided to include preoperative (81 patients) and postoperative (45 patients with a tumor recurrence) CT images. This resulted in a certain bias in the discriminatory power. Based on the independent test data, we obtained a mean accuracy of 0.875 [0.704:1.000] when using preoperative CT images, and a mean accuracy of 0.782 [0.357:1.000] when using postoperative CT images (cases of recurrent tumors). In future studies, only preoperative CT images should be used. It is important to state that a prospective multicentric study is needed for precise study results. In addition to the usually very difficult task of collecting larger study cohorts, an alternative approach could be to use external databases such as the REMBRANDT database (REpository of Molecular BRAin Neoplasia DaTa), published by TCIA (The Cancer Imaging Archive), to validate the trained models. Thirdly, at the time of analysis, the 2016 WHO classification of CNS tumors was still in place. Finally, the authors are aware that the accuracy of machine learning algorithms based on CT images could probably be further improved by using even more advanced algorithms, such as deep learning. However, this was not the focus of our study. In our proof-of-concept study, we were much more interested in investigating the extent to which CT images are suitable for brain diagnostics based on machine learning algorithms, if at all, and how corresponding CT-based models perform compared to models based on MRI images. Despite these limitations, our proof-of-principle study demonstrates that LGGs and HGGs can be successfully differentiated using machine learning algorithms based on CT images. Such methods might reduce invasive diagnostic measures in the future and support immediate treatment planning.

## 6. Conclusions

Our results show that not only MRI scans, but also CT images, can be used for machine learning algorithm-supported brain tumor diagnostics. Using routinely generated contrast-enhanced CT images, our model achieves a mean AUC of 0.903, a mean accuracy of 0.839, a mean sensitivity of 0.807 and a mean specificity of 0.864, based on independent test data. The achieved discriminatory power is comparable to that of corresponding models based on MRI images. However, prospective multicenter studies with larger study cohorts are needed to obtain even more reliable study results in the future. In this context, the discriminatory power that can be achieved with machine learning algorithms based on CT images should be thoroughly investigated, not only in terms of WHO grades, but also in terms of the predictability of important molecular markers, such as IDH or ATRX mutations.

## Figures and Tables

**Table 1:** Demographic and histopathological characteristics of study cohort.

	Training Data	Independent Test Data	Total Data
Number	101	25	126
Low-grade glioma or high-grade glioma (in %)			
LGG	45.54	44.00	45.24
HGG	54.46	56.00	54.76
Gender (in %)			
Male	57.68	58.96	57.94
Female	42.32	41.04	42.06
Mean age (years)	48.86	48.48	48.78

**Table 2:** List of most important features for the three different feature preselection algorithms (in descending order of importance).

	Feature Preselection Algorithm		
Rank	Univariate Analyses	Naive Bayes	Ridge Regression
1	gldm.DependenceEntropy	gldm.DependenceEntropy	glrlm.GrayLevelNonUniformityNormalized
2	glrlm.RunEntropy	glrlm.RunEntropy	Age_at_diagnosis_date
3	glcm.Correlation	glcm.Correlation	shape.Maximum2DDiameterColumn
4	glcm.Idmn	glszm.ZoneEntropy	glcm.Correlation
5	glrlm.GrayLevelNonUniformityNormalized	glrlm.GrayLevelNonUniformityNormalized	glszm.GrayLevelNonUniformityNormalized
6	glszm.ZoneEntropy	firstorder.Maximum	gldm.DependenceEntropy
7	glcm.JointEnergy	glcm.Idmn	firstorder.InterquartileRange
8	shape.Maximum2DDiameterColumn	firstorder.Range	glcm.MCC
9	gldm.LargeDependenceHighGrayLevelEmphasis	firstorder.MeanAbsoluteDeviation	glcm.JointEnergy
10	glszm.GrayLevelNonUniformityNormalized	glcm.ClusterProminence	glcm.Idmn

**Table 3:** Classification results for training data and independent test data, calculated as means of 100 repetitions (100 cycles). Feature preselection was performed using univariate analyses. Naïve Bayes was used for subsequent model construction. Final models include 6 features. Values in brackets: 95% confidence interval.

**Performance Metric**	**Training Data**	**Independent Test Data**
AUC	0.932 [0.883, 0.963]	0.903 [0.757, 1.000]
Accuracy	0.862 [0.752, 0.916]	0.839 [0.701, 0.939]
Sensitivity	0.849 [0.598, 0.935]	0.807 [0.502, 1.000]
Specificity Cohen’s kappa	0.874 [0.791, 0.936] 0.722 [0.490, 0.830]	0.864 [0.714, 1.000] 0.673 [0.390, 0.877]

## Data Availability

The data sets used and/or analyzed during the current study are available from the corresponding author upon reasonable request.
